# Laparoscopic Pyeloplasty in children with Horseshoe Kidney

**DOI:** 10.1590/S1677-5538.IBJU.2016.0042

**Published:** 2017

**Authors:** Paulo Renato Marcelo Moscardi, Roberto Iglesias Lopes, Marcos Figueiredo Mello, Cristovao Machado Barbosa, Bruno Nicolino Cezarino, Lorena Marçalo Oliveira, Francisco Tibor Dénes, Miguel Srougi

**Affiliations:** 1 Divisão de Urologia do Departamento de Cirurgia, Universidade de São Paulo, SP, Brasil

## Abstract

**Introduction:**

Horseshoe kidney occurs in 1 per 400-800 live births and are more frequently observed in males (M:F 2:1). Ureteropelvic junction obstruction (UPJO) is commonly associated with horseshoe kidneys. The variable blood supply, presence of the isthmus and high insertion of the ureter contribute to this problem.

**Case report:**

An asymptomatic 6 year-old boy presented with antenatal hydronephrosis. Ultrasonography and CT scan demonstrated left UPJO associated with a horseshoe kidney. DMSA showed 33% of function on the left side. DTPA showed a flat curve and lack of washout. A left dismembered laparoscopic pyeloplasty was performed after identification of crossing vessels and abnormal implantation of the ureter. After one year, the child is asymptomatic. DTPA demonstrated a good washout curve.

**Results:**

Our cohort consisted of six patients, five males and one female, with a mean age of 6 years (range 6m-17 years) and a mean follow-up of 3 years. Ureteropelvic junction obstruction was more common on the left side. Symptoms appeared only in 34% of the cases. Mean operative time was 198 minutes (range 120-270 minutes). Crossing vessels were common (observed in 50% patients). High implantation of ureter was seen in 67% patients and intrinsic obstruction in 83%. Surgical difficulties were found in two cases. Hospital stay was 4.3 days (3 to 6 days), with only one patient having a mild complication (pyelonephritis). All cases had clinical and radiologic improvement.

**Conclusion:**

Laparoscopic pyeloplasty is safe and feasible in children with UPJO in horseshoe kidneys, with good results and minimal morbidity.

**Available at: http://www.intbrazjurol.com.br/video-section/moscardi_375_375**

